# Facile Synthesis and Optical Properties of CsPbX_3_/ZIF-8 Composites for Wide-Color-Gamut Display

**DOI:** 10.3390/nano9060832

**Published:** 2019-05-31

**Authors:** Shiliang Mei, Bobo Yang, Xian Wei, Hanqing Dai, Zhihao Chen, Zhongjie Cui, Guilin Zhang, Fengxian Xie, Wanlu Zhang, Ruiqian Guo

**Affiliations:** 1 Engineering Research Center of Advanced Lighting Technology, Ministry of Education; Institute for Electric Light Sources, Fudan University, Shanghai 200433, China; meishiliang@fudan.edu.cn (S.M.); 18110720040@fudan.edu.cn (X.W.); 15307130037@fudan.edu.cn (Z.C.); 16210720023@fudan.edu.cn (G.Z.); xiefengxian@fudan.edu.cn (F.X.); fdwlzhang@fudan.edu.cn (W.Z.); 2 Academy for Engineering and Technology, Institute of Future Lighting, Fudan University, Shanghai 200433, China; 18110860060@fudan.edu.cn (B.Y.); 18110860015@fudan.edu.cn (H.D.); 18110860029@fudan.edu.cn (Z.C.)

**Keywords:** perovskite quantum dots, all-inorganic, ZIF-8, composite, wide-color-gamut display

## Abstract

All-inorganic CsPbX_3_ (X = Cl, Br, and I) perovskite quantum dots (QDs), an emerging type of luminescent materials, have drawn extensive attention in recent years. However, the amelioration of their stability is becoming a critical issue. Herein, we present a facile and efficient approach to prepare novel perovskite QDs/metal–organic frameworks (CsPbX_3_/ZIF-8) composites under ambient-atmospheric conditions. The obtained composites exhibit better properties including high photoluminescence (PL) quantum yields (QYs) (41.2% for green and 34.8% for red), narrow-band emission (20 nm for green and 31 nm for red), and enhanced stability in comparison to bare QDs. Furthermore, their application in a remote-type white-light-emitting device was explored and a wide color gamut (~137% of the National Television System Committee standard) was achieved, verifying that these novel luminescent composites have great prospect in backlight display application.

## 1. Introduction

All-inorganic CsPbX_3_ (X = Cl, Br, and I) perovskite quantum dots (QDs) have drawn extensive attention over the past years due to high-fluorescence quantum efficiency, narrow-tunable emission, easy solution-based preparation, and short-carrier lifetime, which endows them with great prospect in light-emitting devices (LEDs), solar cells, low-threshold lasers, photoelectric detectors, and visible-light communication applications [1,2,3,4,5]. However, since the perovskite QDs are ionic crystals and their formation energy is low, their crystal structures are easily damaged under different exposures such as light, heat, moisture, oxygen, etc., which could result in the degradation of photoelectric properties [6]. As this instability greatly limits the practical application of perovskite QDs, the improvement of the stability has become a critical issue.

So far, many efforts have been made to ameliorate the stability of CsPbX_3_ QDs by coating or encapsulating, mainly including two types of routes: One is coating inorganic materials (Al_2_O_3_ [7], SiO_2_ [8,9,10], ZnS [11], etc.) or polymers (trioctylphosphine oxide [12], alkyl phosphate [13], etc.) on the surface of QDs; the other is encapsulating QDs into a matrix such as calcium fluoride [14], polystyrene silicon spheres [15], silicone [16], mesoporous silica [17], zeolite molecular sieves [18], etc. Nevertheless, it seems hard to sustain the excellent optical properties of perovskite QDs when improving their stability, and many polymer coatings are not suitable to high-temperature environments. In addition, some coating or packaging processes are complicated, and some require a glove box absent of oxygen and water. Therefore, it is still urgent to find some proper materials for improving the stability of CsPbX_3_ QDs, and it is also necessary to develop a more convenient and effective route for coating or encapsulating without damaging their optical properties.

Metal–organic frameworks (MOFs), a novel kind of porous, organic–inorganic, hybrid materials, are self-assembled by organic ligands and metal ions and exhibit a series of specific features such as structural diversity, adjustable pore size, and large surface area [19,20,21], and it has been reported in many studies that they can be used as hosts to form novel luminescent composites with various materials such as dye molecules and carbon dots [22,23,24,25]. In contrast, the reports on the perovskite QDs–MOFs composites are relatively few. Zeolite imidazole framework material (ZIF-8), as one type of MOFs with high porosity and good water and thermal stability, has been widely applied in gas separation, catalysis, and sensors [26,27,28]. Based on the properties of CsPbX_3_ QDs and ZIF-8, the fabric of hybrid composites of CsPbX_3_ QDs and ZIF-8 is expected to be helpful for improving the stability of QDs and simultaneously for endowing ZIF-8 with remarkable optical properties.

Herein, we first prepared highly luminescent CsPbX_3_ QDs via a hot-injection approach and then combined them with ZIF-8 to obtain novel CsPbX_3_/ZIF-8 composites. The process was very simple and efficient and was carried out under air conditioning. The as-prepared composites possess better optical properties and stability compared with pristine QDs. Moreover, their application in a remote-type white LED was explored, demonstrating their potential applications in wide-color-gamut display fields.

## 2. Results and Discussion

**Figure 1:**
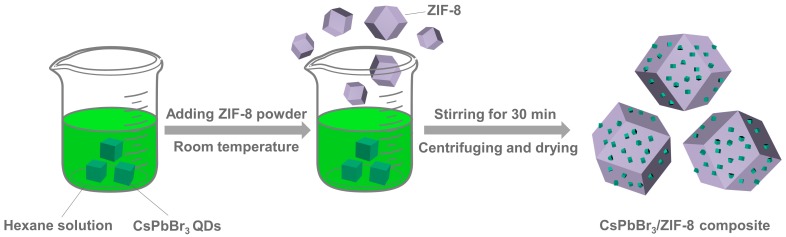
Schematic illustration for the synthesis of the CsPbBr_3_/ZIF-8 composite.

To begin with, CsPbX_3_ QDs with strong green and red emission were synthesized via a typical hot-injection method [29]. The detailed experiments and characterizations are provided in the experimental section. Moreover, a facile and efficient route was employed to obtain CsPbX_3_/ZIF-8 composites at room temperature and normal pressure conditions. The schematic formation process of the representative green CsPbBr_3_/ZIF-8 composite is shown in Figure 1. The ZIF-8 powder was added into the green CsPbBr_3_ QDs/hexane solution, and the mixture was stirred for 30 min. Subsequently, the precipitate was collected via centrifuging (8000 rpm, 10 min). Finally, the CsPbBr_3_/ZIF-8 composite was acquired through drying powders in an oven for 12 h at 50 °C.

**Figure 2:**
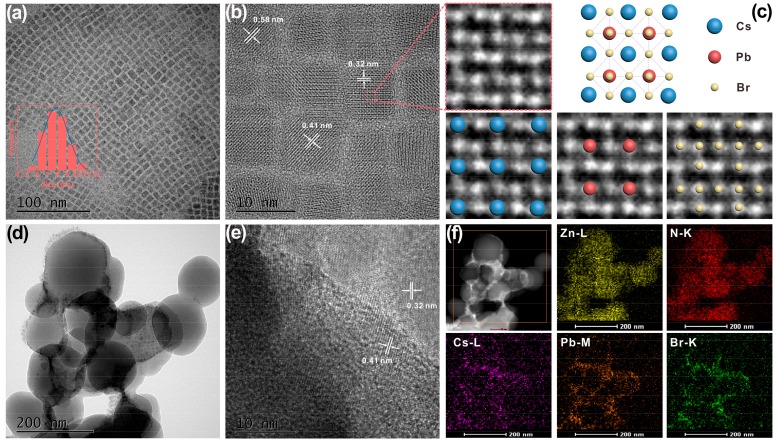
(**a**) TEM (inset: Size distribution) and (**b**) high-resolution TEM (HRTEM) images of monodisperse CsPbBr_3_ quantum dots (QDs). (**c**) Enlarged HRTEM images of CsPbBr_3_ QDs and the corresponding cubic crystal structure. (**d**) TEM and (**e**) HRTEM images of the CsPbBr_3_/ZIF-8 composite. (**f**) EDS elemental mapping images for Zn, N, Cs, Pb, and Br of CsPbBr_3_/ZIF-8 composite.

**Figure 3:**
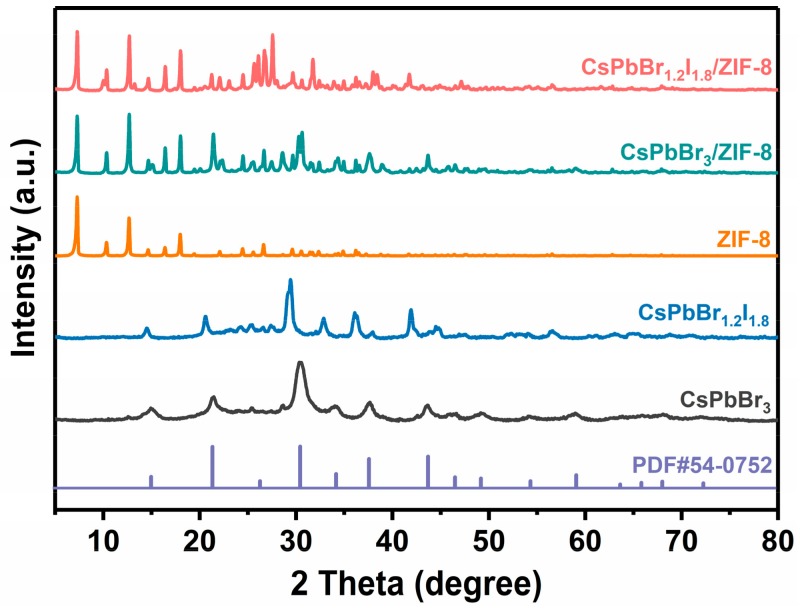
XRD patterns of CsPbX_3_ (X = Br, Br_0.4_I_0.6_) QDs, ZIF-8, and CsPbX_3_/ZIF-8 composites.

The structures of CsPbX_3_ QDs and CsPbX_3_/ZIF-8 composites were investigated by transmission electron microscopy (TEM), X-ray diffraction (XRD), and Fourier transform infrared spectroscopy (FTIR). The TEM image and size distribution of CsPbX_3_ QDs are presented in Figure 2a. It can be observed that the as-prepared CsPbBr_3_ QDs are uniformly distributed with a cubic shape and an average size of 7.9 nm. The high-resolution TEM (HRTEM) image depicted in Figure 2b confirms distinct lattice spaces of 0.32, 0.41, and 0.58 nm that correspond to the d-spacing planes of the (111), (110), and (100) of the cubic CsPbBr_3_ [11,30], respectively. The enlarged HRTEM image in Figure 3c clearly displays the single atom arrangement in CsPbBr_3_ QDs, matching well with the cubic CsPbBr_3_ crystal structure [31]. The TEM image of the obtained CsPbBr_3_/ZIF-8 composite is given in Figure 2d. Both the CsPbBr_3_ QDs and ZIF-8 can be observed. The HRTEM image of composite shown in Figure 2e also verifies the high crystallinity of CsPbBr_3_ QDs with the lattice fringes of 0.32 and 0.42 nm, which is consistent with that of the in-plane lattice spacing of the CsPbBr_3_ phase in Figure 2b. Further analysis of the energy dispersive spectrometer (EDS) elemental mapping of the CsPbBr_3_/ZIF-8 composite is performed by employing Zn, N, Cs, Pb, and Br as detection signals as shown in Figure 2f. It is apparent that the Zn and N from ZIF-8 crystals and the Cs, Pb, and Br elements from CsPbBr_3_ QDs are uniformly dispersed in the selected square area thereby indicating the successful formation of CsPbBr_3_/ZIF-8 composite. The existence of characteristic elements of CsPbBr_3_/ZIF-8 composite can also be confirmed via X-ray photoelectron spectroscopy (XPS) survey (Figure S1). Compared with pristine CsPbBr_3_ QDs, the binding energies of Cs 3d, Pb 4f, and Br 3d in the CsPbBr_3_/ZIF-8 composite (Figure S1c–e) all move to the higher energy side, which may be attributed to the intense interaction of the ions and the imidazole ligand, thus demonstrating an intimate contact between QDs and MOFs. Moreover, according to the atomic ratio of each component derived from the XPS measurements (Table S1), the elemental compositions for CsPbBr_3_ and CsPbBr_3_/ZIF-8 are 20.9% Cs^+^, 16.3% Pb^2+^, 62.8% Br^−^ and 23.1% Cs^+^, 15.8% Pb^2+^, 61.1% Br^−^, respectively, suggesting the invariant cubic crystal structure of CsPbBr_3_ in the composites.

**Figure 4:**
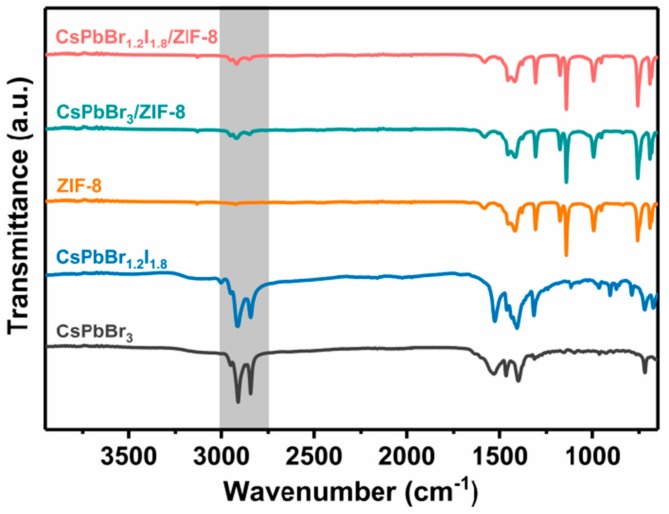
FTIR spectra of CsPbX_3_ (X = Br, Br_0.4_I_0.6_) QDs, ZIF-8, and CsPbX_3_/ZIF-8 composites.

Figure 3 shows the XRD patterns of pure CsPbX_3_ (X = Br, Br_0.4_/I_0.6_) QDs, pure ZIF-8 and CsPbX_3_/ZIF-8 composites. The as-prepared CsPbBr_3_ QDs have mainly several peaks at 15.1°, 21.4°, 30.4°, 34.1°, 37.6°, 43.6°, and 59.1° corresponding to the reflection peaks of (100), (110), (200), (210), (211), (220), and (321) planes of a cubic phase structure (JCPDS No. 54-0752). In contrast, all diffraction peaks of red CsPbBr_1.2_I_1.8_ QDs shift to the small angle direction due to the relatively larger radius of the iodine anion [29]. For the as-prepared CsPbX_3_/ZIF-8 composites, the characteristic peaks of both CsPbX_3_ and ZIF-8 crystal planes can be observed, confirming that ZIF-8 and the perovskite phase exist in the composites. This can also be verified by two additional adsorption bands at 2850 and 2918 cm^−1^ appearing in the CsPBr_3_/ZIF-8 and CsPBr_1.2_I_1.8_/ZIF-8 composites in comparison to pure ZIF-8 crystal (Figure 4), which are assigned to the symmetric and asymmetric stretching vibrations of C–H bonds derived from OLA and OA molecules existing on the surface of perovskite QDs [32].

**Figure 5:**
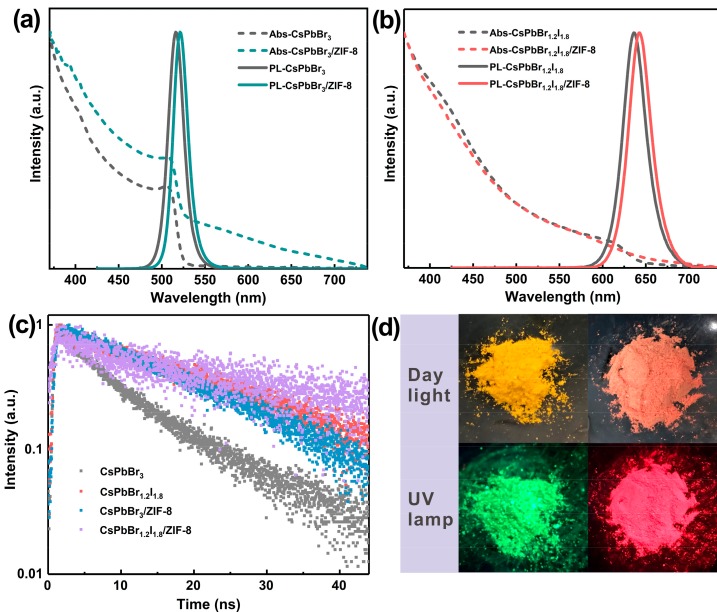
(**a**) UV-vis absorption (dashed line) and photoluminescence (PL) (solid line) spectra of CsPbBr_3_ and CsPbBr_3_/ZIF-8 dispersed in hexane; (**b**) UV-vis absorption (dashed line) and PL (solid line) spectra of CsPbBr_1.2_I_1.8_ and CsPbBr_1.2_I_1.8_/ZIF-8 dispersed in hexane; (**c**) time-resolved PL decay curves of CsPbBr_3_, CsPbBr_1.2_I_1.8_, CsPbBr_3_/ZIF-8, and CsPbBr_1.2_I_1.8_/ZIF-8 powders; (**d**) photographs of CsPbBr_3_/ZIF-8 and CsPbBr_1.2_I_1.8_/ZIF-8 powders under daylight and UV lamp (365 nm).

In addition to the structural characterization discussed above, the optical properties of CsPbX_3_ QDs and CsPbX_3_/ZIF-8 composites were also investigated. Figure 5a,b depicts the ultraviolet-visible (UV-vis) absorption and photoluminescence (PL) spectra of CsPbBr_3_ and CsPbBr_1.2_I_1.8_ QDs and corresponding composites dispersed in hexane, respectively. The emission peaks of CsPbBr_3_ and CsPbBr_1.2_I_1.8_ QDs can be found located at 516 and 636 nm, respectively. In contrast, the emission peaks wavelength of CsPbBr_3_/ZIF-8 and CsPbBr_1.2_I_1.8_/ZIF-8 composites are 521 and 643 nm, respectively, revealing a red shift in comparison to pristine QDs. This red shift may be ascribed to the aggregation effect of pristine QDs in the composites, which is earlier observed for the case of semiconductor QDs [33]. In addition, the full width at half maximum (FWHM) of emission is invariable in the pristine QDs and composite (Table S2). Figure 5c presents the PL decay curves of CsPbX_3_ and CsPbX_3_/ZIF-8 solid powders. These curves can be well fitted by a monoexponential function and the fluorescence lifetimes are 12.55, 18.39, 26.82, and 30.29 ns for CsPbBr_3_, CsPbBr_3_/ZIF-8, CsPbBr_1.2_I_1.8_, and CsPbBr_1.2_I_1.8_/ZIF-8, respectively, as shown in Table S2. The fluorescence lifetimes of composites are found to be longer than that of the pure QDs, which may relate to the reduction of defects on the surface of QDs in the MOFs system. Chirvony V.S. et al. also demonstrated that the long-lived PL decay of lead halide perovskite nanocrystals was due to the aggregation degree of the particles [34]. As a result, the aggregation effect may also result in the prolonged fluorescence lifetimes of composites. Although CsPbBr_3_ and CsPbBr_1.2_I_1.8_ QDs solutions possess high PL quantum yields (QYs) with 84.2% and 73.7%, respectively, the QYs of powders decrease to only 33.6% and 29.1%, since the surface ligands are easily detached during purification and drying process, resulting in the increment of surface defects. The photographs of CsPbBr_3_/ZIF-8 and CsPbBr_1.2_I_1.8_/ZIF-8 powders under daylight and UV lamp (365 nm) are also displayed in Figure 5d. Their PL QYs are 41.2% and 34.8%, respectively, higher than that of the pure QDs, which suggests that ZIF-8 has a certain passivation effect on the surface of CsPbX_3_ QDs, in accordance with the fluorescence lifetime results.

The photostability, thermal stability, moisture resistance, and long-term storage stability test of CsPbBr_3_ QDs and CsPbBr_3_/ZIF-8 composites were also carried out, as shown in Figure S2. After being exposed to the UV lamp (365 nm, 6 W) for 96 h, the CsPbBr_3_/ZIF-8 sustains 43.9% of its original PL intensity, indicating that the ZIF-8 matrix can protect the QDs from photo-oxidation induced by UV light irradiation to some extent (Figure S2a). With heating up, the CsPbBr_3_/ZIF-8 maintains 35.4% of its initial PL intensity at 90 °C (Figure S2b). The moisture resistance test results (Figure S2c) show that the relative PL intensity of CsPbBr_3_/ZIF-8 still retains about 58.1% after 36 h. In addition, the composite preserves 43.4% PL intensity after 30 days (Figure S2d). These results reveal that the composite exhibits improved stability compared with the pure QDs. The combination of ZIF-8 and QDs could reduce the shedding of surface ligands, passivate the surface defects, and slow down the degradation of QDs structures and thus result in the improvement of stability.

**Figure 6:**
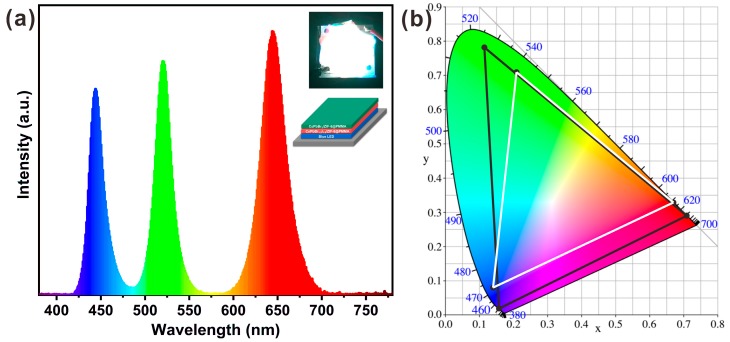
(**a**) PL spectrum of a remote-type white LED fabricated by covering a green CsPbBr_3_/ZIF-8@PMMA film and a red CsPbBr_1.2_I_1.8_/ZIF-8@PMMA film on a blue chip. Inset: Photograph and configuration schematic diagram of the white LED. (**b**) Color gamut of the white LED (black line) compared with the National Television System Committee (NTSC) standard (white line).

As described above, the obtained CsPbX_3_/ZIF-8 composites possess superior properties including high PL QYs, narrow-band emission, and enhanced stability. They were further mixed in PMMA to form green CsPbBr_3_/ZIF-8@PMMA and red CsPbBr_1.2_I_1.8_/ZIF-8@PMMA films (Figure S3), and a remote-type white LED based on the films with a blue-emitting InGaN chip (445 nm) was constructed. The PL spectrum, white-light photograph, and configuration schematic diagram of the as-fabricated LED operated at 20 mA are shown in Figure 6a and three emission peaks at 445, 520, and 644 nm can be distinctly observed. The corresponding Commission International de l’Eclairage (CIE) chromaticity color coordinates is (0.30, 0.30). The color temperature and luminescence efficiency are measured to be 8461 K and 12.85 lm/W. Moreover, the CIE color coordinate triangle covers 137% of the National Television System Committee (NTSC) standard, higher than that using commercial phosphor (85.6% NTSC) [35], Cd-based QDs (104.3% NTSC) [35], and other perovskite QDs (102–130% NTSC) [14,17,36,37,38,39] as color-conversion materials, demonstrating the great prospect in wide-color-gamut backlight display application.

## 3. Conclusions

In summary, we have successfully prepared brightly luminescent CsPbX_3_/ZIF-8 composites via an easy and efficient approach. The obtained composites exhibit superior properties including high PL QYs (41.2% for green and 34.8% for red), narrow-band emission (20 nm for green and 31 nm for red), and enhanced stability. Furthermore, a white LED was fabricated using green CsPbBr_3_/ZIF-8@PMMA and red CsPbBr_1.2_I_1.8_/ZIF-8@PMMA films as color-conversion layers with a blue chip and a wide color gamut with which 137% NTSC was achieved, which reveals that these novel luminescent composites have great prospect in backlight display application.

## 4. Experimental Section

### 4.1. Materials

Cesium carbonate (Cs_2_CO_3_, 99%), lead bromide (PbBr_2_, 99.999%), lead iodide (PbI_2_, 99.999%), octadecene (ODE, 90%), oleylamine (OLA, 90%), and oleic acid (OA, 90%) were purchased from Aladdin. Zinc 2-methylimidazole metal–organic framework (ZIF-8, pore size: 0.34–1.16 nm) was purchased from J & K Chemicals. Polymethyl methacrylate (PMMA, M.W. 35,000) was purchased from Sinopharm (Shanghai, China). All the chemicals were commercially purchased without further purification.

### 4.2. Preparation of Cs-Oleate Solution

A total of 0.8 g Cs_2_CO_3_ was mixed with 30 mL ODE and 2.5 mL OA in a 100 mL flask and degassed at 120 °C under vacuum. The mixture was then heated to 150 °C and kept at this temperature for 30 min until the solution became transparent. The as-prepared Cs precursor was preserved at 100 °C for subsequent injection.

### 4.3. Synthesis of CsPbX_3_ QDs

In order to synthesize green CsPbBr_3_ QDs, 0.132 g PbBr_2_ in 10 mL ODE was loaded in a 50 mL flask and degassed at 120 °C under vacuum for 30 min, followed by injecting 1 mL OLA and 1 mL OA. After being stirred until PbBr_2_ was entirely dissolved, the temperature of the mixture was raised to 150 °C under N_2_ atmosphere and 0.8 mL Cs-oleate solution was rapidly injected. Five seconds later, the flask was immediately immersed in an ice-water bath. The as-prepared stock solution was centrifuged (7000 rpm, 10 min) twice by hexane to collect the precipitates. The obtained QDs were redispersed in hexane/toluene or dried under vacuum for the next characterization. Red CsPbBr_1.2_I_1.8_ QDs were prepared by using 0.0529 g PbBr_2_ and 0.0996 g PbI_2_ following a similar strategy.

### 4.4. Synthesis of CsPbX_3_/ZIF-8 Composites

One hundred milligrams of ZIF-8 powder were added into the as-obtained CsPbX_3_ QDs/hexane solution. The mixture was continuously stirred under ambient atmospheric conditions for 30 min and then centrifuged (8000 rpm, 10 min). So as to remove the surplus QDs, the precipitates were washed by hexane and centrifuged again. Finally, the CsPbX_3_/ZIF-8 composites were dried in a vacuum drying oven for 12 h at 50 °C.

### 4.5. Stability Test

For the photostability test, the as-prepared undried CsPbBr_3_ and CsPbBr_3_/ZIF-8 samples were redissolved in 4 mL toluene and exposed to a UV lamp (365 nm, 6 W) under ambient atmospheric conditions. For the thermal stability test, the obtained CsPbBr_3_ and CsPbBr_3_/ZIF-8 powders were placed on a heating plate and kept at a different temperature for 10 min. For the moisture resistance stability test, the above undried samples were redissolved in the mixed solvent of 4 mL toluene and 0.4 mL deionized water and stored at room temperature. For the long-term storage stability test, the obtained powders were stored in a centrifuge tube under ambient atmospheric conditions for 30 days. During the test procedure, the PL spectrum was measured to record the peak intensity.

### 4.6. Fabrication of the CsPbX_3_/ZIF-8@PMMA Films

The undried CSPbX_3_/ZIF-8 composites mentioned above were redispersed in 2 mL toluene for further utilization. In a 50 mL flask, 1 g PMMA was mixed with 10 mL toluene and heated to 60 °C. After the PMMA was completely dissolved, 2 mL CSPbX_3_/ZIF-8 composites/toluene solution was injected. The mixture was stirred for 1 h to obtain the CsPbX_3_/ZIF-8@PMMA solution. For the fabrication of the CsPbX_3_/ZIF-8@PMMA film, the PMMA solution was first dropped onto a glass substrate placed on a heating plate (60 °C) to form a coating layer. Then the CsPbX_3_/ZIF-8@PMMA solution and a second PMMA layer were coated in sequence after the first layer was dried. Finally, the substrate was dried under ambient condition for 12 h to get a CsPbX_3_/ZIF-8@PMMA film.

### 4.7. Fabrication of a Remote-Type White LED

The as-prepared red CsPbBr_1.2_I_1.8_/ZIF-8@PMMA and green CsPbBr_3_/ZIF-8@PMMA films were pasted on a commercial blue-emitting InGaN chip (445 nm) as color-conversion layers. Fast spectral analysis system (CMS-2S, Inventfine Instrument, Hangzhou, China) and integrating sphere were applied to collect the spectra of the LED at room temperature.

### 4.8. Characterization

UV-vis absorption and PL spectra of samples were measured using a UV-vis spectrophotometer (759S, Shanghai Lengguang, Shanghai, China) and a fluorescence spectrophotometer (F97XP, Shanghai Lengguang, shanghai, China), respectively. The obtained samples were characterized by TEM (JEM-2s100F, JEOL, Tokyo, Japan), XRD (D8 Advance, Bruker, Karlsruhe, Germany), XPS (ESCALAB 250XI, ThermoFisher, Waltham, MA, USA), and FTIR (Nicolet 6700, ThermoFisher, Waltham, MA, USA). The PL decay curves and absolute PL QYs were measured on a fluorescence spectrophotometer (FLS 920, Edinburgh Instruments, Edinburgh, UK).

## Supplementary Materials

The following are available online at https://www.mdpi.com/2079-4991/9/6/832/s1, Figure S1: (a) XPS spectra of CsPbBr_3_ and CsPbBr_3_/ZIF-8 composite and binding energy spectra of (b) Zn 2p, (c) Cs 3d, (d) Pb 4f, and (e) Br 3d for the comparison, Table S1: The atomic ratio of each component in CsPbBr_3_ and CsPbBr_3_/ZIF-8, Table S2: Relevant Parameters of the synthesized CsPbX_3_ and CsPbX_3_/ZIF-8 powders, Figure S2: (a) photostability, (b) thermal stability, (c) moisture resistance and (d) long-term storage stability test of CsPbBr_3_ QDs and CsPbBr_3_/ZIF-8 composites, Figure S3: Schematic representation of the preparation process of CsPbBr_3_/ZIF-8@PMMA film.
