# Supplementary material for: Facile Synthesis and Optical Properties of CsPbX3/ZIF-8 Composites for Wide-Color-Gamut Display
Source: Nanomaterials (Basel). 2019 May 31;9(6):832. doi: 10.3390/nano9060832 (PMC6630813; doi:10.3390/nano9060832)
Supplement: Supplementary file 1 [file nanomaterials-09-00832-s001.pdf]

Supplementary Materials

Facile Synthesis and Optical Properties of $CsPbX_3$/ZIF-8 Composites for Wide-Color-Gamut Display

Shiliang Mei [1], Bobo Yang [2], Xian Wei [1], Hanqing Dai [2], Zhihao Chen [2], Zhongjie Cui [1], Guilin Zhang [1], Fengxian Xie [1], Wanlu Zhang [1] and Ruiqian Guo [1,2,*]

[1] Engineering Research Center of Advanced Lighting Technology, Ministry of Education; Institute for Electric Light Sources, Fudan University, Shanghai 200433, China; meishiliang@fudan.edu.cn (S.M.); 18110720040@fudan.edu.cn (X.W.); 15307130037@fudan.edu.cn (Z.C.); 16210720023@fudan.edu.cn (G.Z.); xiefengxian@fudan.edu.cn (F.X.); fdwlzhang@fudan.edu.cn (W.Z.)

[2] Academy for Engineering and Technology, Institute of Future Lighting, Fudan University, Shanghai 200433, China; 18110860060@fudan.edu.cn (B.Y.); 18110860015@fudan.edu.cn (H.D.); 18110860029@fudan.edu.cn (Z.C.)

* Correspondence: rqguo@fudan.edu.cn; Tel.: +86-021-5566-4588

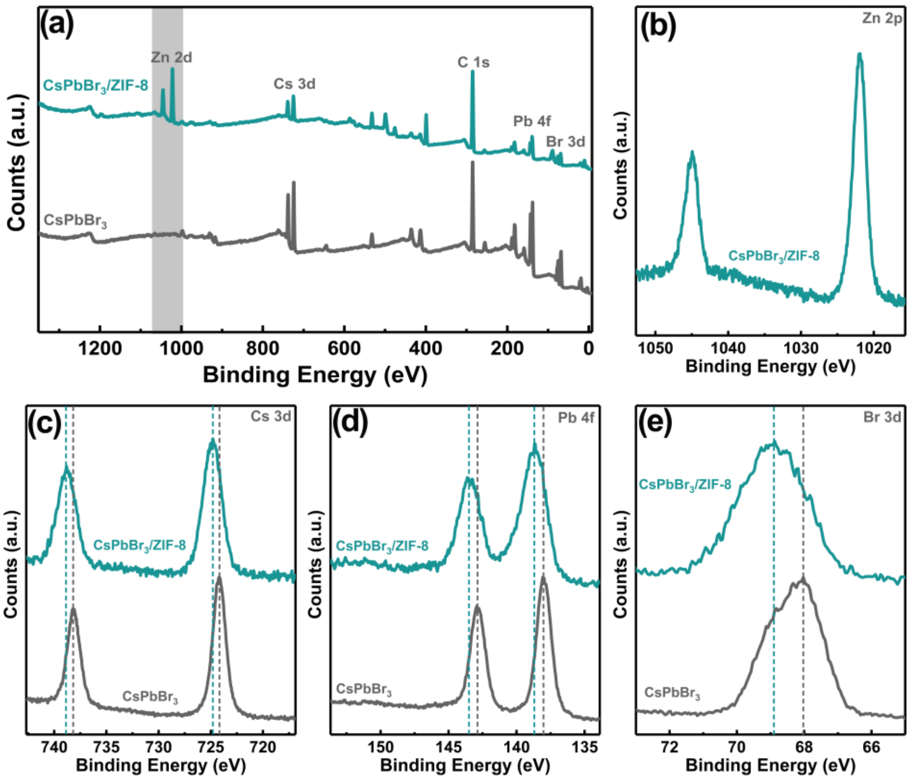

Figure S1. (**a**) XPS spectra of $CsPbBr_3$ QDs and $CsPbBr_3$/ZIF-8 composite and binding energy spectra of (**b**) Zn 2p, (**c**) Cs 3d, (**d**) Pb 4f and (**e**) Br 3d for the comparison.

Table S1. The atomic ratio of each component in $CsPbBr_3$ and $CsPbBr_3$/ZIF-8.

Sample	C/atom. %	Cs/atom. %	Pb/atom. %	Br/atom. %	Zn/atom. %
$CsPbBr_3$	82.72	3.61	2.81	10.86	—
$CsPbBr_3$/ZIF-8	84.99	1.52	1.04	4.02	8.43

Table S2. Relevant parameters of the synthesized $CsPbX_3$ and $CsPbX_3$/ZIF-8 powders.

Sample	λem (nm)	FWHM (nm)	PL QY (%)	PL decay (ns)
$CsPbBr_3$	516	20	33.6%	12.55
$CsPbBr_3$/ZIF-8	521	20	41.2%	18.39
$CsPbBr_{1.2}I_{1.8}$	636	31	29.1%	26.82
$CsPbBr_{1.2}I_{1.8}$/ZIF-8	643	31	34.8%	30.29

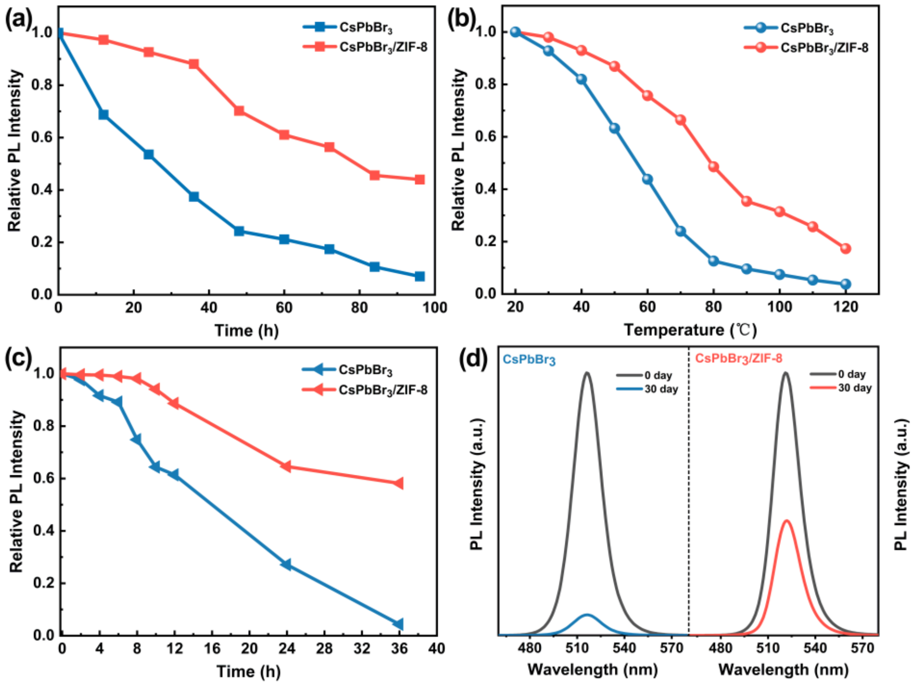

Figure S2. (**a**) Photostability, (**b**) thermal stability, (**c**) moisture resistance and (**d**) long-term storage stability test of $CsPbBr_3$ QDs and $CsPbBr_3$/ZIF-8 composites.

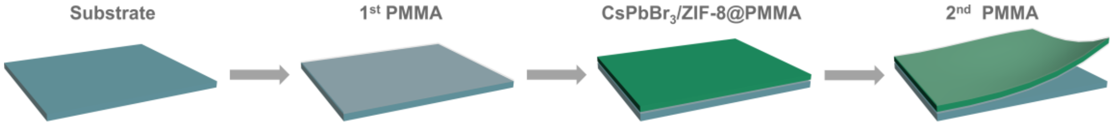

Figure S3. Schematic representation of the preparation process of $CsPbBr_3$/ZIF-8@PMMA film.
